# Supplementary material for: The translational landscape of ground state pluripotency
Source: Nat Commun. 2020 Apr 1;11:1617. doi: 10.1038/s41467-020-15449-9 (PMC7113317; doi:10.1038/s41467-020-15449-9)
Supplement: Supplementary file 1 — Supplementary Information [file 41467_2020_15449_MOESM1_ESM.pdf]

Supplementary Information

The translational landscape of ground state pluripotency

Atlasi et al.

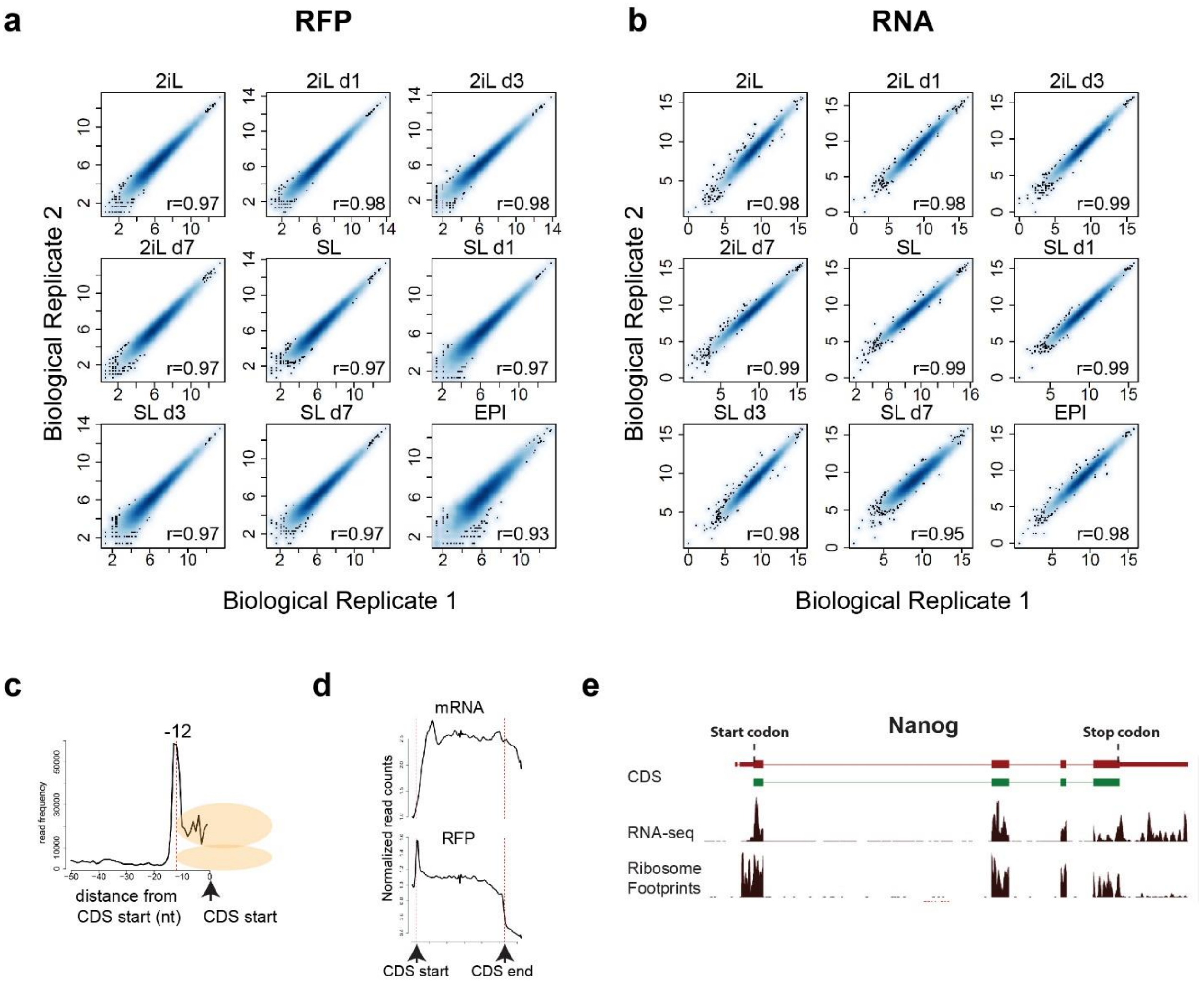

Supplementary Figure 1: Quality control of Ribo-Seq and RNA-seq data

a) Scatter plots showing the correlation between individuals replicates in RFP libraries. r-values represent Pearson correlation coefficient. n=8,546 RFP IDs

b) Scatter plots showing the correlation between individuals replicates in RNA-seq libraries. r-values represent Pearson correlation coefficient. n=11,538 RNA IDs

c) Graph showing the read distribution of the 5'-end of reads in mRNA-Seq and RFP-libraries . For RFP libraries a 12-nt offset was employed to accurately represent the codon at the p-site of the translating ribosomes. Graph was generated using pooled RFP libraries.

d) Graph showing the read coverage at 5´ UTR, coding sequence and 3´ UTR regions in a pool of all prepared RNA-seq and RFP libraries.

e) Genome-browser view of *Nanog*, as a representative example, in RFP and RNA-seq libraries prepared from 2i-ESCs. CDS: coding sequence.

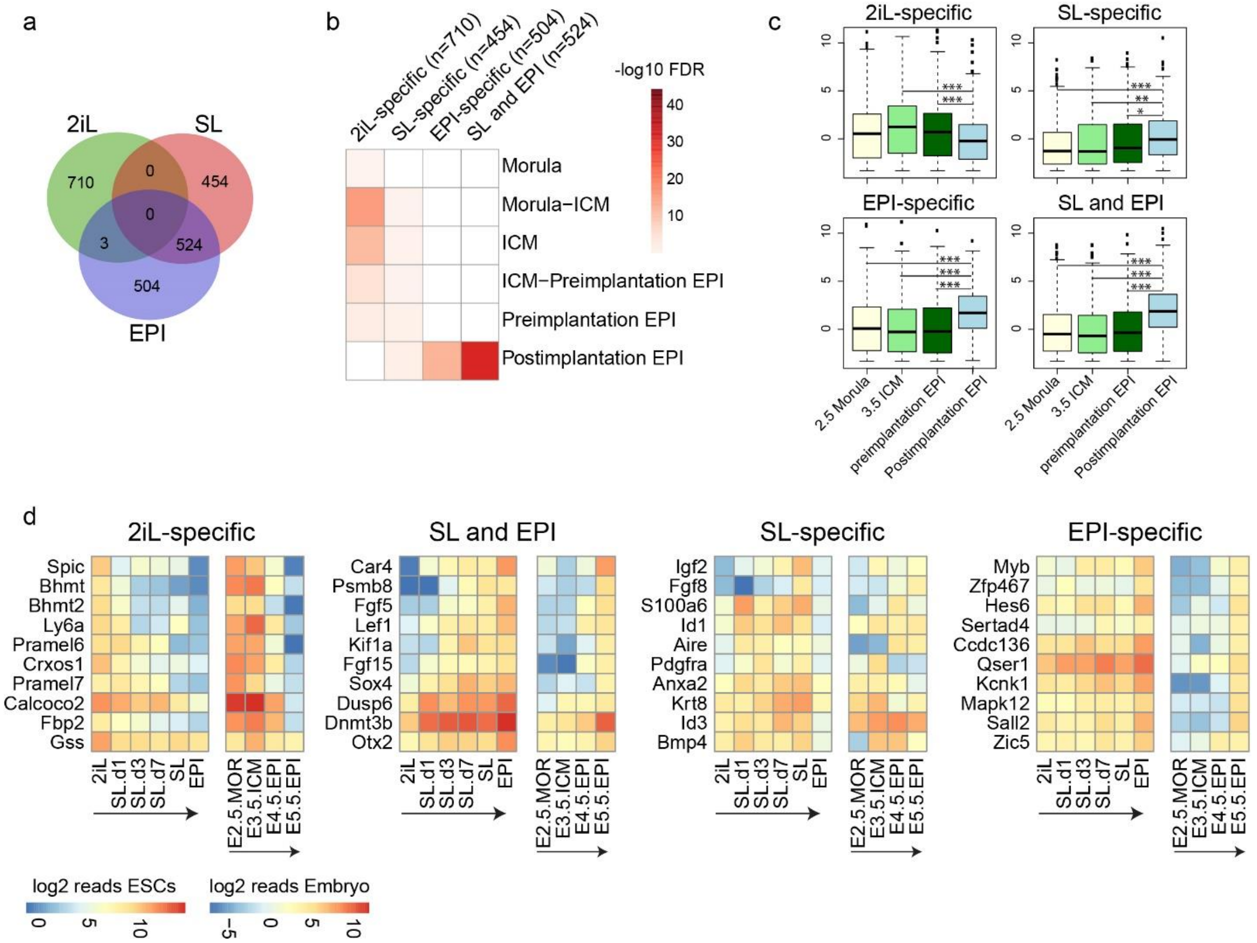

Supplementary Figure 2: Comparison with early embryonic profiles

a) Venn diagram showing the overlap of differentially expressed genes (FC>=3 and DEseq, Benjamini-Hochberg corrected and two-sided P-value <0.05) in 2iL, SL and EPI. N numbers represent mRNAs and are stated in the figure.

b) Heatmap showing enrichment of genes from panel ‘a’ in different stages of embryonic development. N number indicated in the panel represents genes. Two-sided P-values were computed using Hypergeometric tests with Benjamini-Hochberg correction.

c) Box plots showing the expression levels of genes shown in panel ‘b’. Box=25-75th percentile; bar=median; whiskers=5-95th percentile. N numbers represent genes and are stated in the panel ‘b’. Significant difference between groups are marked with asterisks (* p=8.894e-05, ** p=8.999e-07, *** p =4.2e-10); Wilcox test was used to calculate the two-sided P-values.

d) Heat-map showing the DESEQ-normalized mRNA expression values for selected genes during 2iL-to-EPI transition and at different stages of embryonic development.

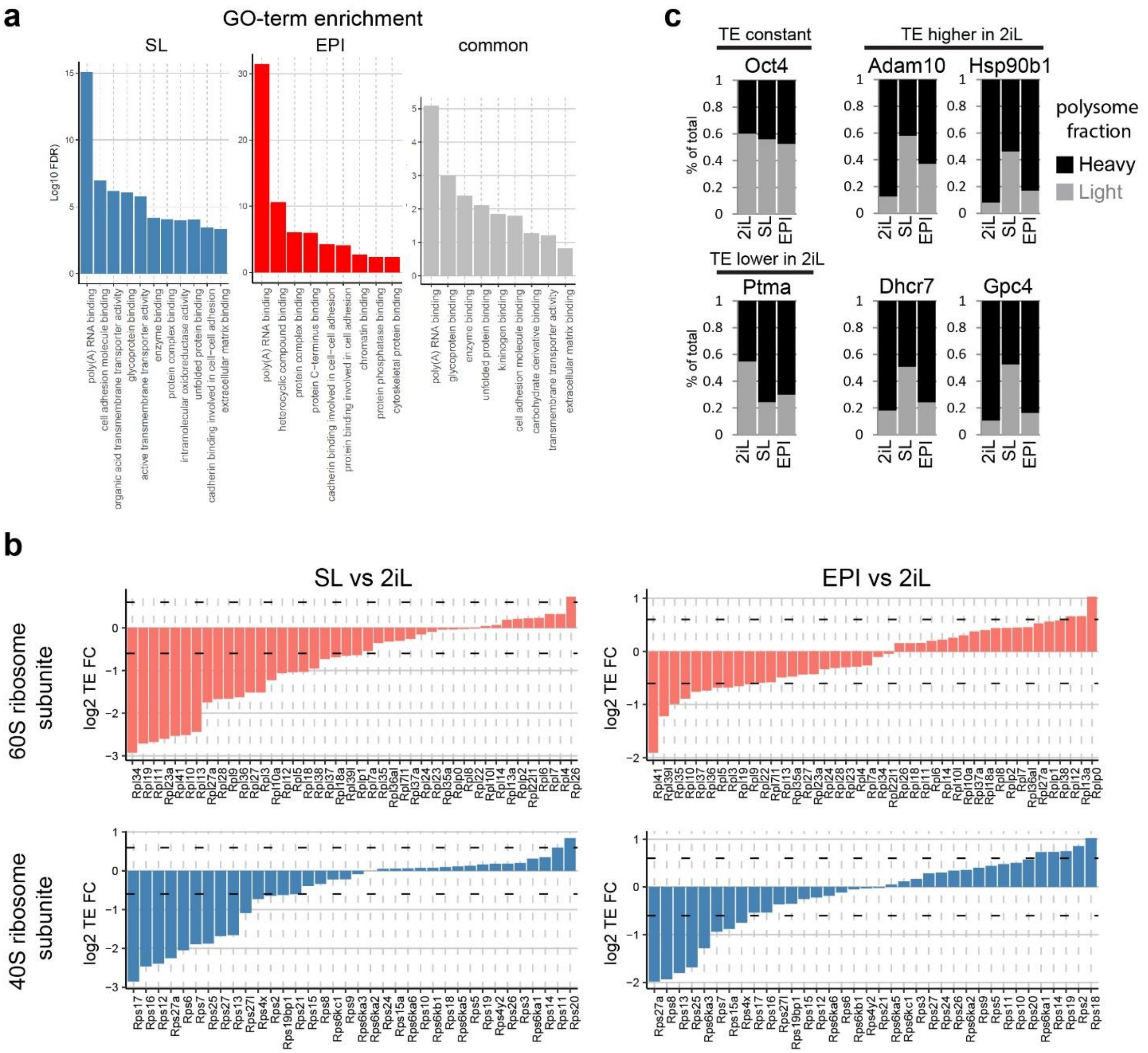

Supplementary Figure 3: GO-term enrichment and selected examples of differentially translated mRNAs

a) GO-term enrichment analysis for SL- , 2iL- or ‘common’-genes. Two-sided P-values were adjusted for multiple testing using Benjamin-Hochberg correction.

b) Graph showing the distribution of selected mRNAs in low- and high-density polysome fractions and in different states of ESCs. The qRT-PCR values represent the mean of two biological replicates.

c) Bar plots showing the TE fold-change of genes encoding the ribosomal proteins in SL or EPI when compared to 2iL. Genes with sufficient number of reads in RNA-seq and RFP-seq (see methods) are shown. Genes were separated based on their distribution in the small or the large ribosomal subunits.

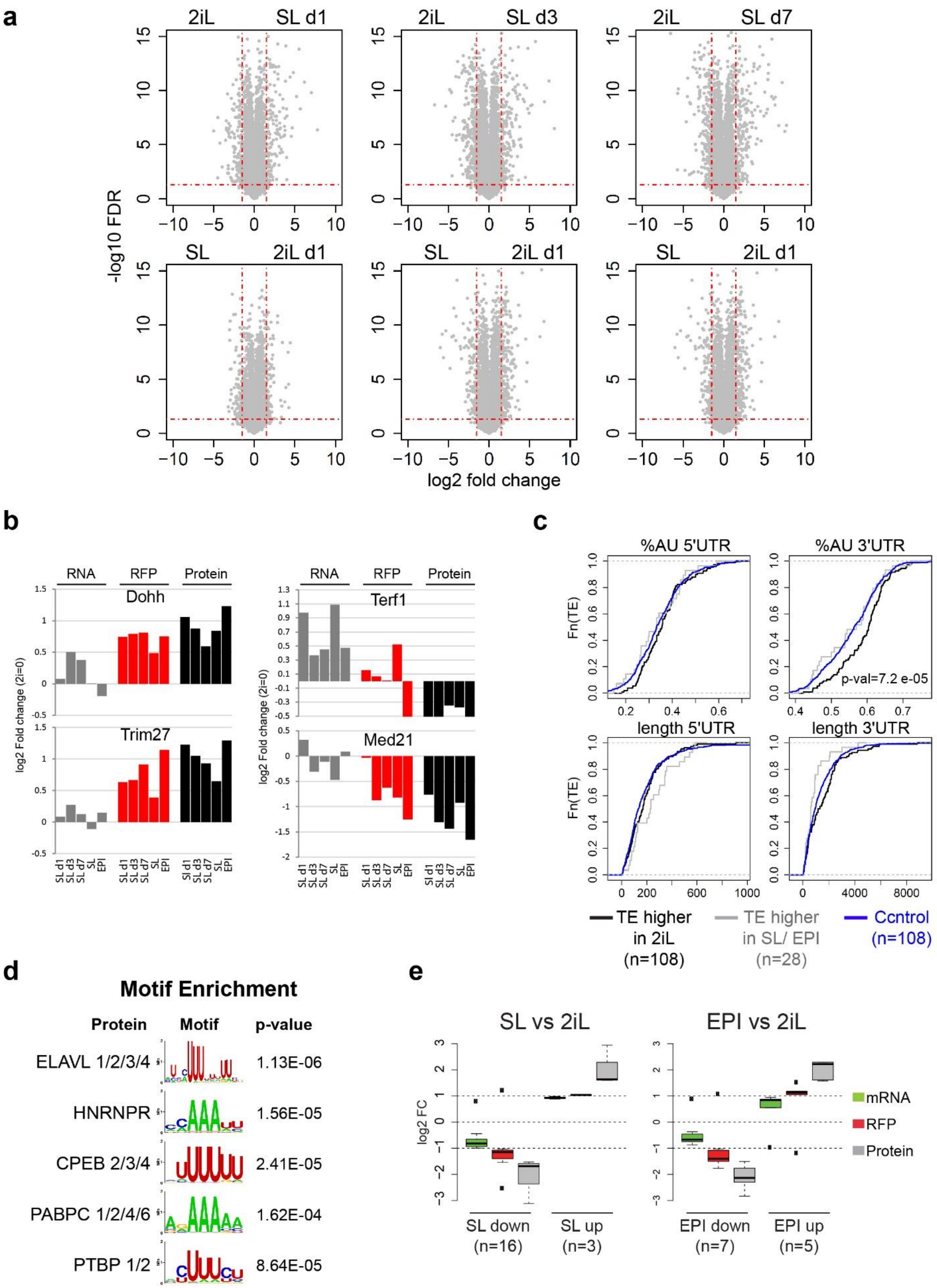

a
2iL SL d1
2iL SL d3
2iL SL d7
SL 2iL d1
SL 2iL d1
SL 2iL d1
-log10 FDR
log2 fold change
b
RNA RFP Protein
Dohh
Trim27
Terf1
Med21
log2 Fold change (2i=0)
SL d1 SL d3 SL d7 SL EPI
c
%AU 5'UTR
%AU 3'UTR
length 5'UTR
length 3'UTR
Fn(TE)
p-val=7.2 e-05
TE higher in 2iL (n=108)
TE higher in SL/ EPI (n=28)
Ccntrol (n=108)
d
Motif Enrichment
Protein Motif p-value
ELAVL 1/2/3/4 1.13E-06
HNRNPR 1.56E-05
CPEB 2/3/4 2.41E-05
PABPC 1/2/4/6 1.62E-04
PTBP 1/2 8.64E-05
e
SL vs 2iL
EPI vs 2iL
log2 FC
mRNA
RFP
Protein
SL down (n=16)
SL up (n=3)
EPI down (n=7)
EPI up (n=5)

Supplementary Figure 4: Structural features and protein-levels of differentially translated mRNAs

a) Volcano plots showing the total proteome quantifications during 2iL-to-SL or SL-to-2iL transition. Values represent the mean of three biological replicates. Differential expression was calculated using a two-sided t-test (FDR <0.05 and FC≥3).

b) Bar plots showing the fold change in RNA, RFP, or protein-levels for 2 representative genes with increased or decreased TE in 2iL ESCs. Changes are shown during 2iL-to-SL and EPI transition.

c) Graphs showing the sequence and structural features of ‘common’-mRNAs that have increased or decreased TEs in 2iL ESCs. Similar number of randomly selected mRNAs were used as control (blue). Wilcox test was used to calculate the P Values.

d) Motif enrichment for RNA-binding protein at the 3´ UTR of ‘common’-genes with increased TE in 2iL ESCs. Two-sided P-values are derived from the hypergeometric tests associated with each motif and were adjusted for multiple testing using Benjamin-Hochberg correction.

e) Box plots showing the RNA, RFP and protein levels for genes that undergo specific translational regulation. Box=25-75th percentile; bar=median; whiskers=5-95th percentile. N numbers represent genes and are stated in the figure.
