# Supplementary material for: The translational landscape of ground state pluripotency
Source: Nat Commun. 2020 Apr 1;11:1617. doi: 10.1038/s41467-020-15449-9 (PMC7113317; doi:10.1038/s41467-020-15449-9)
Supplement: Supplementary file 3 — Reporting Summary [file 41467_2020_15449_MOESM3_ESM.pdf]

natureresearch

Corresponding author(s): Henk Stunnenberg

Last updated by author(s): 1-3-2020

Reporting Summary

Nature Research wishes to improve the reproducibility of the work that we publish. This form provides structure for consistency and transparency in reporting. For further information on Nature Research policies, see Authors & Referees and the Editorial Policy Checklist.

Statistics

For all statistical analyses, confirm that the following items are present in the figure legend, table legend, main text, or Methods section.

n/a	Confirmed	
☐	☒	The exact sample size (n) for each experimental group/condition, given as a discrete number and unit of measurement
☐	☒	A statement on whether measurements were taken from distinct samples or whether the same sample was measured repeatedly
☐	☒	The statistical test(s) used AND whether they are one- or two-sided<br>*Only common tests should be described solely by name; describe more complex techniques in the Methods section.*
☒	☐	A description of all covariates tested
☐	☒	A description of any assumptions or corrections, such as tests of normality and adjustment for multiple comparisons
☒	☐	A full description of the statistical parameters including central tendency (e.g. means) or other basic estimates (e.g. regression coefficient) AND variation (e.g. standard deviation) or associated estimates of uncertainty (e.g. confidence intervals)
☐	☒	For null hypothesis testing, the test statistic (e.g. F, t, r) with confidence intervals, effect sizes, degrees of freedom and P value noted<br>*Give P values as exact values whenever suitable.*
☒	☐	For Bayesian analysis, information on the choice of priors and Markov chain Monte Carlo settings
☒	☐	For hierarchical and complex designs, identification of the appropriate level for tests and full reporting of outcomes
☐	☒	Estimates of effect sizes (e.g. Cohen's d, Pearson's r), indicating how they were calculated

Our web collection on statistics for biologists contains articles on many of the points above.

Software and code

Policy information about availability of computer code

Data collection	For western blot imaging, ImagequantLAS 4000 (GE healthcare life sciences) was used.<br>For chromatin and sequencing library analysis, 2100 expert software (Agilent 2100 Bioanalyzer) was used.
Data analysis	The main data analyses were carried out using R version 3.5.1 on Ubuntu 16.04.5 LTS. Additional R packages used:<br>ggplot2_3.0.0, data.table_1.11.8, DESEQ 1.26.0, XTAIL v.1.1.5<br><br>Command line software used:<br>fastx_toolkit-0.0.14<br>samtools_1.3.1<br>bedtools_v2.20.1<br>bowtie2_2.1.0<br>Python 2.5, Linux 2.6.32-23-generic-pae<br>GMAP version 2014-07-28<br>htseq-count version 0.6.1p1<br>MaxQuant 1.5.1.0<br>Perseus 1.6.5.0<br>Biovenn http://www.biovenn.nl/<br>peakstats.py https://bitbucket.org/simonvh/solexatools/src/master/

For manuscripts utilizing custom algorithms or software that are central to the research but not yet described in published literature, software must be made available to editors/reviewers. We strongly encourage code deposition in a community repository (e.g. GitHub). See the Nature Research guidelines for submitting code & software for further information.

Data

Policy information about availability of data

All manuscripts must include a data availability statement. This statement should provide the following information, where applicable:

- Accession codes, unique identifiers, or web links for publicly available datasets
- A list of figures that have associated raw data
- A description of any restrictions on data availability

RNA-sequencing, RFP-sequencing, Proteomics data that support the findings of this study have been deposited in the Gene Expression Omnibus (GEO) under accession codes GSE133794. The mass spectrometry data have been deposited at the ProteomeXchange Consortium via the PRIDE partner repository with the dataset identifier PXD014528. All other data supporting the findings of this study are available from the corresponding author on reasonable request.

Field-specific reporting

Please select the one below that is the best fit for your research. If you are not sure, read the appropriate sections before making your selection.

☒ Life sciences ☐ Behavioural & social sciences ☐ Ecological, evolutionary & environmental sciences

For a reference copy of the document with all sections, see nature.com/documents/nr-reporting-summary-flat.pdf

Life sciences study design

All studies must disclose on these points even when the disclosure is negative.

Sample size	No statistical methods were used to predetermine sample size.<br>Cell biology experiments and proteomics were performed in three replicates, molecular biology experiments (RNA-seq, RFP-seq) were performed in duplicates as it is widely accepted in high-throughput techniques.
Data exclusions	No data were excluded
Replication	All experiments were repeated at least two times with high reproducibility. High throughput Sequencing of samples was performed once per sample
Randomization	samples were randomly allocated into experimental groups
Blinding	Investigators were not blinded; experiments were objectives all findings are supported by quantitative measurements.

Reporting for specific materials, systems and methods

We require information from authors about some types of materials, experimental systems and methods used in many studies. Here, indicate whether each material, system or method listed is relevant to your study. If you are not sure if a list item applies to your research, read the appropriate section before selecting a response.

Materials & experimental systems

n/a	Involved in the study	
☐	☒	Antibodies
☐	☒	Eukaryotic cell lines
☒	☐	Palaeontology
☒	☐	Animals and other organisms
☒	☐	Human research participants
☒	☐	Clinical data

Methods

n/a	Involved in the study	
☒	☐	ChIP-seq
☒	☐	Flow cytometry
☒	☐	MRI-based neuroimaging

Antibodies

Antibodies used

RNF126 (Abcam, ab234812, 1:500),
BHMT (Abcam, ab96415, 1:500),
NQO1 (Abcam, ab28947, 1:500),
KRAS (Abcam, Ab180772, 1:500),
S100A6 (Abcam, ab134149, 1:1000),
OCT4 (Millipore, MABD76, 1:1000),
ESRRB (Perseus proteomics, PP-H6705-00, 1:1000).
GAPDH (Abcam, Ab8245, 1:1000) and
ACTB (Sigma, A1978, 1:1000)

HRP-rabbit anti mouse: Dako, P0161, Lot:200033538, 1:1000
HRP-swine-anti-rabbit: Dako, P0217, Lot:20040441, 1:3000

Validation	Antibodies were chosen based on previous literature. Validation and quality control is available from the manufacturers using the catalog number of each antibody

Eukaryotic cell lines

Policy information about cell lines

Cell line source(s)	E14 mouse cells were purchased from ATCC
Authentication	E14 cells were purchased from ATCC and were not authenticated
Mycoplasma contamination	All cell lines were negative for mycoplasma
Commonly misidentified lines (See ICLAC register)	No cell lines used in this study were found in the database of commonly misidentified cell lines that is maintained by ICLAC and NCBI Biosample.
