# Supplementary material for: The translational landscape of ground state pluripotency
Source: Nat Commun. 2020 Apr 1;11:1617. doi: 10.1038/s41467-020-15449-9 (PMC7113317; doi:10.1038/s41467-020-15449-9)
Supplement: Supplementary file 4 — Description of Additional Supplementary Files [file 41467_2020_15449_MOESM4_ESM.pdf]

Description of Additional Supplementary Files

Supplementary Data 1: List of all datasets generated in this study

Supplementary Data 2: List of RFP, RNA and TE values in different states of pluripotency and during 2iL-SL-EPI transition

Supplementary Data 3: list of total proteome and differentially expressed proteins in different states of pluripotency and during 2iL-SL-EPI transition

Supplementary Data 4: Integrative list of RFP, RNA and protein values in different states of pluripotency and during 2iL-SL-EPI transition. Genes that were uniquely assigned at RNA, RFP and protein levels and in all three ESC-states are shown.

Supplementary Data 5: List of differentially expressed genes that are grouped based on their specific transcriptional, translational or post-translational regulation downstream of GSK- or MEK-inhibition

Supplementary Data 6: List of employed primers
