# Supplementary figures and images for: The translational landscape of ground state pluripotency
Source: Nat Commun. 2020 Apr 1;11:1617. doi: 10.1038/s41467-020-15449-9 (PMC7113317; doi:10.1038/s41467-020-15449-9)

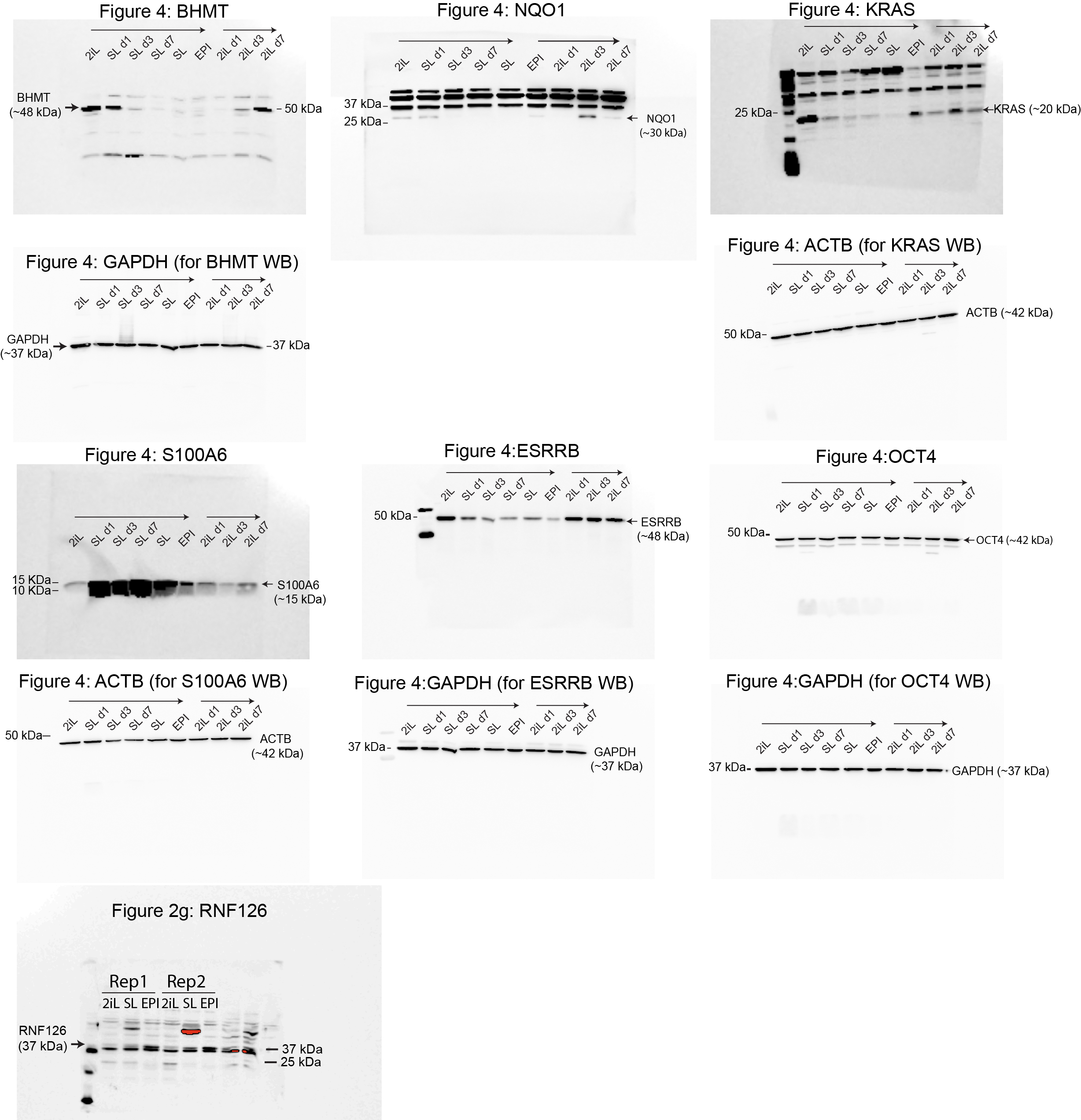

Supplement: Supplementary file 11 — Source Data [file 41467_2020_15449_MOESM11_ESM.zip › western blot image.jpg]
